# Eosinophilic Airway Inflammation in Patients with Stable Biomass Smoke- versus Tobacco Smoke-Associated Chronic Obstructive Pulmonary Disease

**DOI:** 10.5696/2156-9614-9.24.191209

**Published:** 2019-11-27

**Authors:** Lalita Fernandes, Shraddha Rane, Suresh Mandrekar, Anthony Menezes Mesquita

**Affiliations:** 1 Department of Pulmonary Medicine, Goa Medical College, Goa, India; 2 Department of Pathology, Goa Medical College, Goa, India

**Keywords:** biomass smoke, chronic obstructive pulmonary disease, COPD, eosinophils, inflammation, pathogenesis, small airway

## Abstract

**Background.:**

Chronic obstructive pulmonary disease (COPD) is an inflammatory disease with predominant involvement of neutrophils, macrophages and CD8+ lymphocytes. Eosinophilic airway inflammations are reported in stable state and during acute exacerbations of tobacco smoke-associated COPD (TS-COPD). Women exposed to biomass fuel smoke are known to have eosinophils in sputum. However, little is known about the sputum cellular inflammatory profile in biomass fuel smoke-associated COPD (BMS-COPD). We therefore aimed to compare the sputum cellular inflammatory profile in tobacco smoke- and biomass smoke-associated COPD.

**Methods.:**

The study was conducted in a tertiary care hospital in Goa, India. A total of 113 patients with stable COPD reporting to the outpatient pulmonary clinic were recruited. All participants were ≥ 40 years of age. Sputum induction studies were performed by the method of Pizzichini *et al.* after baseline subject characterization. Significant eosinophilia was defined as induced sputum eosinophils ≥ 3%.

**Results.:**

There were 85 TS-COPD and 28 BMS-COPD patients. The mean age [standard deviation (SD)] was 64.7 (7.8) and 63.0 years (8.3), p = 0.32 in TS and BMS-COPD, respectively. Eighteen subjects (21.1%) were female smokers. The smoking pack-year median [interquartile range (IQR)] was 36 (20, 58) and hour-years of biomass smoke exposure mean (SD) was 192.4 (61). The TS-COPD and BMS-COPD cases showed a post-bronchodilator forced expiratory volume in one second (FEV1%) mean (SD) of 57.9 (17.1), and 62.6 (19.4), p= 0.22, respectively. Both groups had similar symptoms and severity of disease. Induced sputum total cell count per gram of sputum × 10^6^ mean (SD) was 3.05 (1.53) for TS-COPD, and 2.55(1.37) for BMS-COPD p=0.12. The neutrophils % mean (SD) was 86.4 (16.5) and 87.9 (10.2), p = 0.64; eosinophils % median (IQR) was 2.5 (1, 10) and 8 (2, 12.8), p = 0.07; lymphocytes % median (IQR) was 0 (0, 0.75) and 0 (0, 1) p = 0.13; macrophages % median (IQR) was 2.5 (0.75, 5.7) and 1 (0, 4.7) p = 0.13; and significant eosinophilia (eosinophils ≥3%) was 42 (49.4%) and 20 (71%), p=0.04, for TS-COPD and BMS-COPD, respectively.

**Conclusions.:**

For similar severity of disease and clinical symptoms, significant eosinophilic inflammation was observed in stable BMS-COPD, while both groups had similar neutrophilic inflammation.

**Participant Consent.:**

Obtained.

**Ethics Approval.:**

The study was approved by the Institutional Ethics Committee of the Goa Medical College, Goa, India.

**Competing Interests.:**

The authors declare no competing financial interests.

## Introduction

Chronic obstructive pulmonary disease (COPD) is a common disorder and is currently the third leading cause of death worldwide.^1^ Chronic obstructive pulmonary disease is defined as an inflammatory disease caused mainly by exposure to tobacco and biomass fuel smoke apart from exposure to other noxious particles and gases. The predominant cells of inflammatory response are neutrophils, macrophages and CD8 + lymphocytes.^2^,^3^ Chronic inhalation of cigarette smoke can modulate both innate and adaptive immune responses.^4^ In more severe cases, adaptive response develops in the lungs and is linked with additional activation of IgG-producing B cells. About 20–40% of patients with tobacco smoke-associated COPD (TS-COPD) have eosinophils in induced sputum during acute exacerbations and during clinical stability.^5–8^ Inflammation is the central feature of stable COPD, causing activation and recruitment of infiltrating inflammatory cells. Identification of the type of cellular inflammation in stable COPD, whether neutrophilic or eosinophilic, helps to explore better management strategies for COPD. Studies have shown that sputum eosinophilia predicts benefit from prednisone in smokers with COPD, short term response to inhaled corticosteroid, prevention of exacerbations of COPD and response to inhaled corticosteroids with long acting β_2_ agonists.^9–12^

Cigarette smoke is the major risk factor for COPD in developed countries, while in developing countries (both low- and middle-income) biomass fuel smoke exposure is the main risk factor, especially in women. Three billion people worldwide are exposed to biomass smoke and 90% of the rural population use biomass fuel for cooking, heating and lighting.^13^ Recent quantitative computed tomography of thorax studies in COPD have shown that tobacco smoke-associated COPD (TS-COPD) is more emphysema predominant, while biomass smoke-associated COPD (BMS-COPD) has less emphysema, but similar levels of small airway inflammation.^14^,^15^ Women exposed to biomass fuel smoke have eosinophils in induced sputum, however the sputum cellular inflammatory profile in biomass smoke-associated COPD has been less extensively studied.^16^

The present study aimed to determine the induced sputum cellular inflammatory profile in patients with TS- and BMS-associated COPD. We hypothesized that BMS-COPD will have significant eosinophilia in induced sputum compared to TS-COPD.

**Abbreviations**
*BMS*: Biomass smoke
*COPD*: Chronic obstructive pulmonary disease
*FEV_1_*: Forced expiratory volume in one second
*TS*: Tobacco smoke

## Methods

This was a cross-sectional study conducted at the Chest Diseases Hospital of a tertiary care teaching center in Goa, India. The Chest Diseases Hospital is a free, walk-in center for patients suffering from pulmonary diseases. A total of 113 patients were enrolled with stable COPD reporting to the outpatient pulmonary clinic: 85 TS-COPD and 28 BMS-COPD cases. Significant biomass smoke exposure for women occurred while cooking. The tobacco smoke-associated COPD subjects did not report significant exposure to biomass fuel smoke as they did not personally partake in the cooking process. All subjects were ≥ 40 years of age. Chronic obstructive pulmonary disease was diagnosed by the Global Initiative for Chronic Obstructive Lung Disease guidelines with a post-bronchodilator forced expiratory volume in one second (FEV_1_)/forced vital capacity < 70% in a stable clinical state.^17^ We excluded patients with dual exposure to tobacco and biomass smoke, acute exacerbation of COPD within 4 weeks of enrollment, use of systemic steroids within the last 6 weeks of enrollment and any other respiratory disease, including asthma. The study was approved by the Institutional Ethics Committee of the Goa Medical College, and all participants gave written informed consent.

Spirometry was performed as per American Thoracic Society/European Respiratory Society guidelines.^18^ Respiratory symptoms and risk factors for COPD were evaluated using the Burden of Obstructive Lung Disease core and biomass questionnaire.^19^ Exposure to biomass smoke was reported in hour-years, which is the product of the average daily number of hours the patient spends cooking and the number of years cooking using biomass fuel. The cumulative exposure to tobacco smoke was quantified as pack-years.

### Sputum induction and processing

Sputum was induced by the method described by Pizzichini *et al*.^20^ The standard operating procedure was followed by trained personnel. If post bronchodilator FEV_1_ was < 800 ml, the subject was nebulized with normal saline (0.9%) and if FEV_1_ was > 800 ml then hypertonic saline in increasing concentration was used (3%, 4% and 5%). Nebulization was carried out with an ultrasonic nebulizer (Smart Care) with an output set to 0.6–0.8 ml/min. The subject inhaled the mist through the mouthpiece for 5 minutes with tidal breathing. The procedure was stopped when selected sputum weight was > 0.8 g. Post-saline inhalation spirometry was performed to assess the safety of the procedure. A patient was considered safe to discharge if FEV_1_ was within 90% of baseline pre-bronchodilator FEV_1_. Sputum was collected on ice and processed immediately at 4°C as per standard guidelines. The selected sputum was mixed with 0.1% dithiothreitol in the ratio 1:4, vortexed for 15 seconds and then rocked on a bench rocker for 15 minutes. To stop the effect of dithiothreitol, four times volume of Dulbecco phosphate buffer saline was added and filtered. Proportions of squamous cell, cell viability and total cell count were obtained using the trypan blue exclusion method. The cell pellet was re-suspended in Dulbecco phosphate buffer saline to 0.5×10^6^ cells per ml and cytospun. Slides were stained for differential count and evaluated by an experienced pathologist blinded to patients' clinical details.

### Statistical analysis

Statistical analysis was performed using the Statistical Package for the Social Sciences program (SPSS) version 24 (IBM Corp, SPSS Inc, Chicago, IL). Normality of data was assessed using the Shapiro-Wilk test. Continuous variables are presented either as mean [standard deviation (SD)] or as median and interquartile range (IQR) when normality assumptions of the distribution were not satisfied. Categorical variables are presented as percentages. Independent Student's t-test, chi-square test and Mann-Whitney U test were applied to study the differences between the two groups. A p value of < 0.05 was considered to be statistically significant.

## Results

Of the 113 subjects, 85 were TS-COPD cases and 28 were BMS-COPD cases, with no significant age difference between the two groups, with a mean age of 64.7 (7.8) and 63.0 years (8.3), respectively; p = 0.32. A total of 18 (21.1%) were female smokers. Most of the participants came from different parts of Goa, had mostly rural backgrounds, came from low- and middle-income groups and belonged to farming communities. Both groups had similar severity of disease; post bronchodilator FEV_1_% was 57.9 (17.1) for TS-COPD cases, and 62.6 (19.4) for BMS-COPD cases (p = 0.22). Bronchodilator responsiveness was noted in 30 (35.2%) of TS-COPD cases, and one (3.5%) of the BMS-COPD cases, p=0.001. Significant eosinophilia (eosinophils ≥3%) 42 (49.4%), 20 (71%), p=0.04 was noted in TS-COPD and BMS-COPD cases, respectively. There was no difference in the Global Initiative for Chronic Obstructive Lung Disease severity of disease and significant eosinophilia; p= 0.09. All subjects provided adequate sputum samples. Baseline patient characteristics are presented in Table 1, while differential cell count in induced sputum is presented in Table 2.

**Table 1:** Baseline Characteristics of Chronic Obstructive Pulmonary Disease Patients

	**TS-COPD**	**BMS-COPD**	**P**
n	85	28	
Age mean (SD)	64.7 (7.8)	63.0 (8.3)	0.32
Males (n)	67	0	
Females (n)	18	28	
BMI mean (SD)	19.1 (3.4)	20.8 (4.2)	0.03^*^
Cough (n (%))	20 (23.5)	3(10.7)	0.14
Phlegm (n (%))	42 (49.4)	8 (28.5)	0.05
Wheeze (n (%))	61 (71.7)	20 (71.4)	0.97
Smoking (pack-years) median (IQR)	36 (20, 58)	0	
Biomass smoke exposure (hour-years) mean (SD)	0	192.4 (61.0)	
FEV_1_ pre mean (SD)	1.06 (0.41)	0.805 (0.28)	0.25
FEV_1_ pre % mean (SD)	50.7(16.4)	55.2(18.8)	0.23
FEV_1_ post mean (SD)	1.21 (0.44)	0.91 (0.28)	0.001
FEV_1_ post % mean (SD)	57.9(17.1)	62.6(19.4)	0.22
FEV_1_/FVC post mean (SD)	53.6(9.19)	56.25 (10.67)	0.21
Bronchodilator responsiveness (n (%))	30 (35.2)	1 (3.5)	0.001

Abbreviations: pre, pre bronchodilator; post, post bronchodilator; n, number; FVC, forced vital capacity; IQR, interquartile range, SD, standard deviation

^*^ Statistically significant

**Table 2:** Differential Cell Count of Induced Sputum in Tobacco Smoke-Associated and Biomass Smoke-Associated—Chronic Obstructive Pulmonary Disease Patients

	**TS-COPD**	**95% CI**	**BMS-COPD**	**95% CI**	**P**
Total cells per gram of sputum × 10^6^ mean (SD)	3.05 (1.53)	(2.7, 3.3)	2.55 (1.37)	(2.0, 3.0)	0.12^+^
Median (IQR)	2.64(1.91, 3.82)	–	2.18 (1.67, 2.89)	–	
Neutrophils % mean (SD)	86.4(16.5)	(82, 90)	87.9 (10.2)	(84,91)	0.64^+^
Median (IQR)	91.0 (84.5, 96.1)	–	90.0 (81.7, 95.8)	–	
Eosinophils % median (IQR)	2.5 (1, 10)	–	8 (2,12.8)	–	0.07^++^
Mean (SD)	6.73 (9.9)	(4.5, 8.8)	8.30 (7.34)	(5.4, 11)	
Lymphocytes % median (IQR)	0 (0, 0.75)	–	0 (0,1)	–	0.13^++^
Mean (SD)	1.34 (5.2)	(0.2, 2.4)	0.98 (1.88)	(0.25, 1.7)	
Macrophages % median (IQR)	2.5 (0.75, 5.7)	–	1 (0,4.7)	–	0.13^++^
Mean (SD)	5.47 (11.8)	(2.9, 8.0)	2.71 (3.48)	(1.3, 4.0)	
Eosinophils ≥3% n(%)	42 (49.4)	(36, 60)	20 (71)	(53, 89)	0.04^*+++^

Abbreviations: n, number; CI, confidence interval; IQR, interquartile range, SD, standard deviation

+ Students t test,

++ Mann Whitney U test,

+++ Chi Square test.

## Discussion

We have identified for the first time that patients with BMS-COPD have significant eosinophilia in induced sputum; 71% compared to 49.4% in TS-COPD. Significant eosinophilia is defined as induced sputum eosinophils ≥ 3%, which has clinical significance. Eosinophils are derived from the bone marrow under the influence of granulocyte monocyte colony stimulating factor, interleukin-3 and interleukin-5 and are released into circulation from where they lie in the gastrointestinal tract and thymus. The normal eosinophil count in induced sputum of a healthy non-smoker is < 1.1%.^21^

Biomass smoke alters innate immune responses and the three main classes of cell receptors such as the Toll-like receptors, the scavenger receptors and the transient receptor channels, which have the ability to transduce signals initiated after biomass smoke exposure.^22^ After being exposed to dry dung smoke, rats showed increased levels of inflammatory cells in the perivascular, peribronchial and parenchymal region, as well as in the bronchoalveolar lavage fluid.^23^,^24^ A few studies showed increased pulmonary macrophages, neutrophils, eosinophils, lymphocytes, mast cells, interleukin-6, and tumor necrosis factor α in women exposed to biomass smoke compared to other fuels.^25^ Eosinophilic airway inflammation is seen in TS-COPD patients and a count of ≥ 3% is associated with good corticosteroid response in these patients.^26–29^ We observed significant eosinophilia in BMS-COPD cases. The role of eosinophils in the pathophysiology of TS-COPD is less understood, however there is similar pattern of expression of type 2 mediators in the airway of patients with COPD and asthma.^30^ Similarly, the role of eosinophils in BMS-COPD is less studied. Further research is necessary to understand eosinophilia in BMS-COPD.

The normal neutrophil count in a healthy non-smoker is 40–60%, and both groups showed high total neutrophil count even in a stable state. Neutrophilia and raised total cells per gram in sputum is an indicator of infection and is effectively managed with antibiotics. A study by Bafadhel *et al.* showed that sputum neutrophil percentage correlated with bacteria in patients with TS-COPD.^31^

Understanding immuno-pathophysiology of BMS-COPD will provide an informed basis for rational drug use in eosinophilic and neutrophilic inflammation. As biomass smoke-associated COPD has eosinophilic inflammation, response to inhaled corticosteroids may be studied using randomized controlled trials. Currently, BMS-COPD and TS-COPD are similarly managed. In addition, as a large number of people living in low- and middle-income countries use biomass fuel and have COPD, identification of significant eosinophilia in such patients may be helpful in disease management.

The present study was limited due to a small sample size. Studies with adequate sample size would highlight the level of eosinophilia in induced sputum, and the role of the eosinophil and therapeutics of inhaled corticosteroids in BMS-COPD. In addition, all BMS-COPD subjects were female as cooking activities are specifically performed by women in India. However, it is unlikely that gender has an effect on the level of eosinophils in COPD, as eosinophil count was raised in both groups, but BMS-COPD showed significant eosinophilia of ≥ 3%.

## Conclusions

Biomass smoke-related COPD is associated with significant eosinophilia in induced sputum. Further studies are needed to understand the pathophysiology for informed drug use and reduction of morbidity and mortality associated with this disease.

## References

[1] Lozano R, Naghavi M, Foreman K, Lim S, Shibuya K, Aboyans V, Abraham J, Adair T, Aggarwal R, Ahn SY, Alvarado M, Anderson HR, Anderson LM, Andrews KG, Atkinson C, Baddour LM, Barker-Collo S, Bartels DH, Bell ML, Benjamin EJ, Bennett D, Bhalla K, Bikbov B, Bin Abdulhak A, Birbeck G, Blyth F, Bolliger I, Boufous S, Bucello C, Burch M, Burney P, Carapetis J, Chen H, Chou D, Chugh SS, Coffeng LE, Colan SD, Colquhoun S, Colson KE, Condon J, Connor MD, Cooper LT, Corriere M, Cortinovis M, de Vaccaro KC, Couser W, Cowie BC, Criqui MH, Cross M, Dabhadkar KC, Dahodwala N, De Leo D, Degenhardt L, Delossantos A, Denenberg J, Des Jarlais DC, Dharmaratne SD, Dorsey ER, Driscoll T, Duber H, Ebel B, Erwin PJ, Espindola P, Ezzati M, Feigin V, Flaxman AD, Forouzanfar MH, Fowkes FG, Franklin R, Fransen M, Freeman MK, Gabriel SE, Gakidou E, Gaspari F, Gillum RF, Gonzalez-Medina D, Halasa YA, Haring D, Harrison JE, Havmoeller R, Hay RJ, Hoen B, Hotez PJ, Hoy D, Jacobsen KH, James SL, Jasrasaria R, Jayaraman S, Johns N, Karthikeyan G, Kassebaum N, Keren A, Khoo JP, Knowlton LM, Kobusingye O, Koranteng A, Krishnamurthi R, Lipnick M, Lipshultz SE, Ohno SL, Mabweijano J, MacIntyre MF, Mallinger L, March L, Marks GB, Marks R, Matsumori A, Matzopoulos R, Mayosi BM, McAnulty JH, McDermott MM, McGrath J, Mensah GA, Merriman TR, Michaud C, Miller M, Miller TR, Mock C, Mocumbi AO, Mokdad AA, Moran A, Mulholland K, Nair MN, Naldi L, Narayan KM, Nasseri K, Norman P, O'Donnell M, Omer SB, Ortblad K, Osborne R, Ozgediz D, Pahari B, Pandian JD, Rivero AP, Padilla RP, Perez-Ruiz F, Perico N, Phillips D, Pierce K, Pope CA, Porrini E, Pourmalek F, Raju M, Ranganathan D, Rehm JT, Rein DB, Remuzzi G, Rivara FP, Roberts T, De León FR, Rosenfeld LC, Rushton L, Sacco RL, Salomon JA, Sampson U, Sanman E, Schwebel DC, Segui-Gomez M, Shepard DS, Singh D, Singleton J, Sliwa K, Smith E, Steer A, Taylor JA, Thomas B, Tleyjeh IM, Towbin JA, Truelsen T, Undurraga EA, Venketasubramanian N, Vijayakumar L, Vos T, Wagner GR, Wang M, Wang W, Watt K, Weinstock MA, Weintraub R, Wilkinson JD, Woolf AD, Wulf S, Yeh PH, Yip P, Zabetian A, Zheng ZJ, Lopez AD, Murray CJ, AlMazroa MA, Memish ZA (2012). Global and regional mortality from 235 causes of death for 20 age groups in 1990 and 2010: a systematic analysis for the Global Burden of Disease Study 2010. Lancet [Internet].

[2] Brusselle GG, Joos GF, Bracke KR (2011). New insights into the immunology of chronic obstructive pulmonary disease. Lancet [Internet].

[3] Barnes PJ (2008). Immunology of asthma and chronic obstructive pulmonary disease. Nat Rev Immunol [Internet].

[4] Sopori M (2002). Effects of cigarette smoke on the immune system. Nat Rev Immunol [Internet].

[5] Saetta M, Di Stefano A, Maestrelli P, Turato G, Ruggieri MP, Roggeri A, Calcagni P, Mapp CE, Ciaccia A, Fabbri LM (1994). Airway eosinophilia in chronic bronchitis during exacerbations. Am J Respir Crit Care Med [Internet].

[6] Bafadhel M, McKenna S, Terry S, Mistry V, Reid C, Haldar P, McCormick M, Haldar K, Kebadze T, Duvoix A, Lindblad K, Patel H, Rugman P, Dodson P, Jenkins M, Saunders M, Newbold P, Green RH, Venge P, Lomas DA, Barer MR, Johnston SL, Pavord ID, Brightling CE (2011). Acute exacerbations of chronic obstructive pulmonary disease: identification of biologic clusters and their biomarkers. Am J Respir Crit Care Med [Internet].

[7] Singh D, Kolsum U, Brightling CE, Locantore N, Agusti A, Tal-Singer R (2014). Eosinophilic inflammation in COPD: prevalence and clinical characteristics. Eur Respir J [Internet].

[8] Kim VL, Coombs NA, Staples KJ, Ostridge KK, Williams NP, Wootton SA, Devaster JM, Aris E, Clarke SC, Tuck AC, Bourne SC, Wilkinson TM (2017). Impact and associations of eosinophilic inflammation in COPD: analysis of the AERIS cohort. Eur Respir J [Internet].

[9] Pizzichini E, Pizzichini MM, Gibson P, Parameswaran K, Gleich GJ, Berman L, Dolovich J, Hargreave FE (1998). Sputum eosinophilia predicts benefit from prednisone in smokers with chronic obstructive bronchitis. Am J Respir Crit Care Med [Internet].

[10] Brightling CE, McKenna S, Hargadon B, Birring S, Green R, Siva R, Berry M, Parker D, Monteiro W, Pavord ID, Bradding P (2005). Sputum eosinophilia and the short term response to inhaled mometasone in chronic obstructive pulmonary disease. Thorax [Internet].

[11] Wedzicha JA, Calverley PM, Seemungal TA, Hagan G, Ansari Z, Stockley RA (2008). The prevention of chronic obstructive pulmonary disease exacerbations by salmeterol/fluticasone propionate or tiotropium bromide. Am J Respir Crit Care Med [Internet].

[12] Siddiqui SH, Guasconi A, Vestbo J, Jones P, Agusti A, Paggiaro P, Wedzicha JA, Singh D (2015). Blood eosinophils: a biomarker of response to extrafine beclomethasone/formoterol in chronic obstructive pulmonary disease. Am J Respir Crit Care Med [Internet].

[13] Salvi S, Barnes PJ (2010). Is exposure to biomass smoke the biggest risk factor for COPD globally?. Chest. [Internet].

[14] Camp PG, Ramirez-Venegas A, Sansores RH, Alva LF, McDougall JE, Sin DD, Pare PD, Muller NL, Silva CI, Rojas CE, Coxson HO (2014). COPD phenotypes in biomass smoke- versus tobacco smoke-exposed Mexican women. Eur Respir J [Internet].

[15] Fernandes L, Gulati N, Fernandes Y, Mesquita AM, Sardessai M, Lammers JJ, Mohamed Hoesein FA, Ten Hacken NH, van den Berge M, Galban CJ, Siddiqui S (2017). Small airway imaging phenotypes in biomass- and tobacco smoke-exposed patients with COPD. ERJ Open Res [Internet].

[16] Dutta A, Roychoudhury S, Chowdhury S, Ray MR (2013). Changes in sputum cytology, airway inflammation and oxidative stress due to chronic inhalation of biomass smoke during cooking in premenopausal rural Indian women. Int J Hyg Environ Health [Internet].

[17] Vestbo J, Hurd SS, Agustí AG, Jones PW, Vogelmeier C, Anzueto A, Barnes PJ, Fabbri LM, Martinez FJ, Nishimura M, Stockley RA, Sin DD, Rodriguez-Roisin R (2013). Global strategy for the diagnosis, management, and prevention of chronic obstructive pulmonary disease: GOLD executive summary. Am J Respir Crit Care Med [Internet].

[18] Miller MR, Hankinson J, Brusasco V, Burgos F, Casaburi R, Coates A, Crapo R, Enright P, van der Grinten CP, Gustafsson P, Jensen R, Johnson DC, MacIntyre N, McKay R, Navajas D, Pedersen OF, Pellegrino R, Viegi G, Wanger J (2005). Standardisation of spirometry. Eur Respir J [Internet].

[19] Buist AS, Vollmer WM, Sullivan SD, Weiss KB, Lee TA, Menezes AM, Crapo RO, Jensen RL, Burney PG (2005). The Burden of Obstructive Lung Disease Initiative (BOLD): rationale and design. COPD [Internet].

[20] Pizzichini E, Pizzichini MM, Efthimiadis A, Evans S, Morris MM, Squillace D, Gleich GJ, Dolovich J, Hargreave FE (1996). Indices of airway inflammation in induced sputum: reproducibility and validity of cell and fluid-phase measurements. Am J Respir Crit Care Med [Internet].

[21] Belda J, Leigh R, Parameswaran K, O'Byrne PM, Sears MR, Hargreave FE (2000). Induced sputum cell counts in healthy adults. Am J Respir Crit Care Med [Internet].

[22] Olloquequi J, Silva OR (2016). Biomass smoke as a risk factor for chronic obstructive pulmonary disease: effects on innate immunity. Innate Immun [Internet].

[23] Dogan OT, Elagoz S, Ozsahin SL, Epozturk K, Tuncer E, Akkurt I (2011). Pulmonary toxicity of chronic exposure to tobacco and biomass smoke in rats. Clinics (Sao Paulo) [Internet].

[24] Hu G, Zhou Y, Hong W, Tian J, Hu J, Peng G, Cui J, Li B, Ran P (2013). Development and systematic oxidative stress of a rat model of chronic bronchitis and emphysema induced by biomass smoke. Exp Lung Res [Internet].

[25] Banerjee A, Mondal NK, Das D, Ray MR (2012). Neutrophilic inflammatory response and oxidative stress in premenopausal women chronically exposed to indoor air pollution from biomass burning. Inflammation [Internet].

[26] Domagala-Kulawik J, Maskey-Warzechowska M, Kraszewska I, Chazan R (2003). The cellular composition and macrophage phenotype in induced sputum in smokers and ex-smokers with COPD. Chest [Internet].

[27] Kolsum U, Damera G, Pham TH, Southworth T, Mason S, Karur P, Newbold P, Singh D (2017). Pulmonary inflammation in patients with chronic obstructive pulmonary disease with higher blood eosinophil counts. J Allergy Clin Immunol [Internet].

[28] Brightling CE, Monteiro W, Ward R, Parker D, Morgan MD, Wardlaw AJ, Pavord ID (2000). Sputum eosinophilia and short-term response to prednisolone in chronic obstructive pulmonary disease: a randomised controlled trial. Lancet [Internet].

[29] Leigh R, Pizzichini MM, Morris MM, Maltais F, Hargreave FE, Pizzichini E (2006). Stable COPD: predicting benefit from high-dose inhaled corticosteroid treatment. Eur Respir J [Internet].

[30] Bafadhel M, McCormick M, Saha S, McKenna S, Shelley M, Hargadon B, Mistry V, Reid C, Parker D, Dodson P, Jenkins M, Lloyd A, Rugman P, Newbold P, Brightling CE (2012). Profiling of sputum inflammatory mediators in asthma and chronic obstructive pulmonary disease. Respiration [Internet].

[31] Bafadhel M, Haldar K, Barker B, Patel H, Mistry V, Barer MR, Pavord ID, Brightling CE (2015). Airway bacteria measured by quantitative polymerase chain reaction and culture in patients with stable COPD: relationship with neutrophilic airway inflammation, exacerbation frequency, and lung function. Int J Chron Obstruct Pulmon Dis [Internet].

